# Expression of basic fibroblast growth factor is associated with poor outcome in non-Hodgkin's lymphoma

**DOI:** 10.1038/sj.bjc.6600330

**Published:** 2002-06-05

**Authors:** I Pazgal, Y Zimra, C Tzabar, E Okon, E Rabizadeh, M Shaklai, O Bairey

**Affiliations:** Institute of Hematology and Felsenstein Medical Research Center, Rabin Medical Center, Beilinson Campus, Petah Tiqva, 49100, Israel; Institute of Pathology, Rabin Medical Center, Beilinson Campus, Petah Tiqva, 49100, Israel; Sackler Faculty of Medicine, Tel Aviv University, Tel Aviv, Israel

**Keywords:** basic fibroblast growth factor, basic fibroblast growth factor receptor, immunohistochemistry, non-Hodgkin's lymphoma, microvessel count

## Abstract

It is now clear that angiogenesis and angiogenesis factors are important in the pathogenesis of haematological malignancies. High pretreatment levels of serum basic fibroblast growth factor have been shown to be associated with poor prognosis in patients with non-Hodgkin's lymphoma. The aim of this study was to evaluate whether non-Hodgkin's lymphoma cells express basic fibroblast growth factor and/or its receptor (fibroblast growth factor receptor-1) and whether basic fibroblast growth factor expression correlates with basic fibroblast growth factor serum levels, intratumoral microvessel density, and patient outcome. We measured basic fibroblast growth factor by enzyme-linked immunosorbent assay in sera taken from 58 patients with non-Hodgkin's lymphoma before treatment and in 19 of them also after treatment. Pathological specimens at diagnosis were evaluated by immunohistochemistry staining using polyoclonal antibody against factor-VIII-related antigen, basic fibroblast growth factor and fibroblast growth factor receptor-1 to determine the expression of the microvessel count and basic fibroblast growth factor and fibroblast growth factor receptor-1. The lymphoma specimens demonstrated positive staining for basic fibroblast growth factor (in 23%) and fibroblast growth factor receptor-1 (in 58.5%). The patients who expressed basic fibroblast growth factor had a significantly worse progression-free and overall survival than those who did not (*P*=0.003 and *P*=0.03 respectively), while patients expressing fibroblast growth factor receptor-1 were less likely to achieve complete remission than those lacking the receptor (33% *vs* 65% , *P*=0.047). There was no correlation of basic fibroblast growth factor staining with either serum basic fibroblast growth factor levels or microvessel count. Basic fibroblast growth factor serum levels did not change significantly after treatment These results suggest that non-Hodgkin's lymphoma specimens express basic fibroblast growth factor and its receptor (fibroblast growth factor receptor-1) and this expression is associated with poor patient outcome.

*British Journal of Cancer* (2002) **86**, 1770–1775. doi:10.1038/sj.bjc.6600330 www.bjcancer.com

© 2002 Cancer Research UK

## 

Angiogenesis is necessary for the growth of solid tumours and the dissemination of malignant cells (Folkman, 1990). Recent evidence also points to a role of angiogenesis and angiogenesis-related molecules in haematological malignancies (Mangi and Newland, 2000). Tumours promote angiogenesis by secreting growth factors that stimulate endothelial cell migration and capillary proliferation. Angiogenic activity is regulated by the balance between positive and negative angiogenic regulators (Iruela-Arispe and Dvorak, 1997). Basic fibroblast growth factor (bFGF), an 18- to 24-kD polypeptide, serves as a key positive angiogenic regulator *in vivo* (Bikfalvi *et al*, 1997). It also stimulates endothelial cell proliferation *in vitro* and regulates the expression of several molecules thought to mediate critical steps in the angiogenesis process (Basilico and Moscatelli, 1992). bFGF is mainly produced by cells of mesenchymal origin. T cells, macrophages, and granulocytes also have the capacity to produce bFGF (Salven *et al*, 1999). It is normally bound to heparin and heparan sulphate proteoglycans in the extracellular matrix, particularly in basement membranes, around vessels and stromal cells. It binds to a family of four distinct, high-affinity tyrosine kinase receptors, designated FGFR-1–4.

Until recently, measurement of intratumoral microvessel density by immunocytochemistry appeared to be the most reliable method of assessing angiogenic activity (Toi *et al*, 1996). An alternative method for evaluating angiogenic activity is the determination of the levels of each angiogenic factor in tumour tissues and serum (Poon *et al*, 2001). There are very few studies of angiogenesis in non-Hodgkin's lymphoma (NHL). Ribatti and his group (Ribatti *et al*, 1996) assessed the microvessel count with factor VIII staining in 88 patients with NHL and 15 with benign lymphadenopathies. Their count covered the whole specimens. They found that angiogenesis is more intense in the stroma of B-NHL than of benign lymphadenopathies, and increases in more aggressive tumours. In lymphadenopathies and follicular subtypes of NHL, very few vessels were observed within follicles, and numerous in uninvolved tissue between the follicles. By contrast, all diffuse subtypes of NHL showed vessels throughout the tumour tissue. Salven *et al* (1999) measured serum bFGF in 160 newly diagnosed patients with NHL and found that a high pretreatment serum bFGF was associated with poor overall survival.

In the present study, we measured serum bFGF concentration before and after treatment in patients with NHL. We also conducted a biopsy study to determine the expression of bFGF and its receptor FGFR-1 and the microvessel count (MVC) in biopsies taken at diagnosis. Finally, we evaluated the prognostic significance of bFGF and FGFR-1 expression in NHL patients.

## PATIENTS AND METHODS

### Patients

Serum bFGF concentration was measured in 58 adult patients with NHL diagnosed and treated in the Division of Hematology, Rabin Medical Center, Beilinson Campus from 1997 to 1999. Approval was obtained from the local ethics committee. Serum was taken at the time of diagnosis, before lymphoma treatment was administered. In 19 patients, serum bFGF concentration was also measured after 2–3 cycles of chemotherapy.

The clinical and pathological characteristics of the patients are shown in Table 1

**Table 1:**
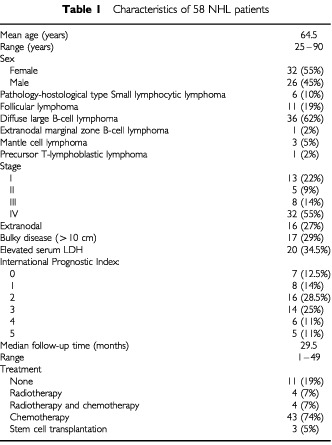
Characteristics of 58 NHL patients

.There were 26 male (45%) and 32 females (55%) of mean age 64.5 years (range 25–90).The histological types according to the WHO classification (Harris *et al*, 1999) were as follows: small lymphocytic lymphoma, six patients (10%); follicular lymphoma, 11 patients (19%); diffuse large B-cell lymphoma (DLBCL), 36 patients (62%); extranodal marginal zone B-cell lymphoma, one patient (2%); mantle cell lymphoma, three patients (5%), and precursor T-lymphoblastic lymphoma, 1 patient (2%). Clinical staging at diagnosis was done according to the Ann Arbor classification system: 13 (22%) patients had stage I, five (9%) stage II, eight (14%) stage III, and 32 (55%) stage IV. Sixteen specimens (27%) were of extranodal origin and the rest were of nodal origin. Seventeen patients (29%) had bulky disease at presentation (largest diameter ⩾10 cm) and 20 (34.5%) had elevated serum lactate dehydrogenase (LDH) levels at diagnosis. The International Prognostic Index (IPI) (The International Non-Hodgkin's Prognostic Factor Project, 1993) was determined in 56 patients. The score was 0 in seven patients (12.5%); one in eight patients (14%); two in 16 patients (28.5%); three in 14 patients (25%); four in six patients (11%), and five in five patients (9%).

Eleven patients did not receive any treatment. Four patients received only radiotherapy and another four received radiation combined with chemotherapy. Low-grade lymphomas were usually treated with chlorambucil alone or combined with prednisone if symptomatic. Intermediate-grade lymphomas were usually treated with CHOP (cyclophosphamide, doxorubicin, vincristine and prednisone) or another anthracycline-containing combination chemotherapy regimen. Three patients underwent stem cell transplantation, two autologous and one allogeneic.

Median follow-up time was 29.5 months (range 1–49). During follow-up, 13 patients died.

### Methods

#### Serum bFGF immunoassay

Peripheral venous blood samples were collected in empty tubes, centrifuged at 2000 **g** for 10 min, and stored at −70°C. The level of bFGF in serum was determined with a commercial quantitative sandwich enzyme immunoassay technique (Quantikine R; R&D Systems, Minneapolis, MN, USA). The system uses a solid-phase monoclonal antibody and an enzyme-linked polyclonal antibody raised against recombinant human bFGF. All analyses and calibrations were performed in duplicate.

#### Immunohistochemistry for factor-VIII-related antigen, bFGF and FGFR-1

All specimens were archival material of biopsies taken at the time of lymphoma diagnosis that had been fixed in neutral-buffered formalin and embedded in paraffin by routine methods. Paraffin-embedded tumour specimens were cut into 4-μm thick sections and mounted on adhesive-coated glass slides. Deparaffinisation and hydration were performed through xylene and graded alcohol series. The slides were washed in distilled water and heated twice in citrate buffer (pH 6.0) in a pressure cooker that was placed in a microwave oven for 15 and 5 min at 700 W to retrieve the antigenicity. Endogenous peroxidase activity was then blocked by incubation for 15 min at room temperature with H_2_O_2_ (3%) in methanol followed by washing with phosphate-buffered saline (PBS). The slides were then incubated with one of the following primary antibodies: (1) rabbit anti-human, anti-factor-VIII-related antigen (F8RA), (Dako, CA, USA) at a dilution of 1 : 1000 for 45 min at room temperature; (2) rabbit anti-human, anti-bFGF polyclonal antibody (Biotechnology, Inc; Santa Cruz, CA, USA) at a dilution of 1 : 500 for 45 min at room temperature; (3) rabbit anti-human, anti-FGFR-1 polyclonal antibody (Biotechnology, Inc.) at a dilution of 1 : 800 for 45 min at room temperature. The primary antibodies against F8RA and FGFR-1 were detected by the Strept A-B immunoperoxidase staining universal kit (LSAB^+^ Kit, Dako) according to the manufacturer's instructions. The primary antibody against bFGF was detected by the DAKO EnVision™+ System. This system is based on HRP-labelled polymer that does not contain avidin or biotin and therefore eliminates nonspecific staining from endogenous avidin-biotin activity, which was very high with the Dako LSAB^+^ kit. Diaminobenzidine was used as chromogen, and incubation was conducted for 5 min at room temperature. Sections were counterstained in haematoxylin. A negative control omitting the primary antibody was used in each experiment. For positive control of bFGF, we stained specimens of normal breast, in which bFGF is known to be localised in the myoepithelial cells (Yiangou *et al*, 1997). For positive control of FGFR-1, we stained specimens of breast carcinoma which is known to display FGFR-1 expression in neoplastic cells (Blanckaert *et al*, 1998). The lymphoid cells in reactive lymph node showed positive staining for bFGF and negative staining for FGFR-1, which did stain the blood vessels.

#### Immunohistological scoring

The areas of highest protein expression evident at low-power scanning were taken for analysis. Staining was considered negative only after careful examination of the entire tissue section under high power (×1000). Quantitation of the number of positive tumour cells was performed simultaneously by two investigators (I Pazgal, O Bairey) blinded to the clinical outcome. A double-headed light microscope was used to score at least 500 cells in high power fields. In cases in which the investigators disagreed, the immunohistochemical staining was repeated, and a third reviewer (E Okon) scored the slides in a blinded fashion. Specimens that contained >10% immunostained tumour cells were defined as immunopositive; those with ⩽10% were defined as immunonegative.

#### Microvessel count

Microvessel count (MVC) was assessed with a light microscope. The whole tumour section was scanned at a low magnification (×100), and the area of the most intense vascularisation (hot spot) was determined. Any brown-staining endothelial cell or endothelial-cell cluster that was clearly separated from adjacent microvessels, tumour cells and connective tissue elements was considered a single countable microvessel. The presence of a vessel lumen was not necessary for a structure to be defined as a microvessel. Vessels within sclerotic areas were not included. Individual microvessel counts were made on a ×200 field (0.785 mm^2^ per field). Quantitation of the number of microvessels was performed simultaneously and independently by two investigators (O Bairey, I Pazgal) blinded to the clinical outcome. The mean number of microvessels per sample was calculated and was taken as the MVC for each case.

#### Statistical analysis

Statistical analysis was performed using an SPSS statistical software program (SPSS Inc. Chicago, IL, USA). Either *t*-test or χ^2^ analysis (Fisher exact test) was used for statistical comparison of the clinical and laboratory characteristics and bFGF serum levels or bFGF and its receptor expression and MVC. Analysis of Variance (ANOVA) was used to compare bFGF levels between different clinical groups. Progression-free survival (PFS) was calculated from date of diagnosis to date of disease progression, relapse or death. Survival and PFS were computed according to the product-limit method of Kaplan-Meier from the date of diagnosis. The log rank test was used to compare survival rates between different subgroups of patients. The relative influence of different variables on survival was studied by multivariate survival analysis using stepwise Cox regression.

## RESULTS

### Serum bFGF in patients at diagnosis and after treatment

Mean serum bFGF concentration in 11 healthy volunteers was 2.5±3.8 pg ml^−1^ (range 0–10.5). Serum bFGF concentrations in 58 patients with NHL ranged from undetectable to 28.0 pg ml^−1^ (mean 5.3±5.6 pg ml^−1^). There were no significant differences in serum bFGF concentration by NHL grade, histologic type, stage of disease, IPI index, or treatment protocol. There was also no association between serum bFGF level and patient age, sex and LDH levels. Patients with bulky NHL had significantly higher levels of bFGF (>10 pg ml^−1^) than patients with non-bulky disease (29.4% and 7.7% respectively, *P*=0.046); no correlation with either overall survival or PFS was found.

In 19 patients, serum bFGF was also assayed after 2–3 cycles of chemotherapy. Mean bFGF level at that time was 9.6 pg ml^−1^ (range 0–21.8). There was no correlation between serum bFGF level at diagnosis and after treatment, nor was there a significant change in bFGF levels or a specific trend in patients responding or not responding to treatment.

### Microvessel count (MVC)

**Figure 1:**
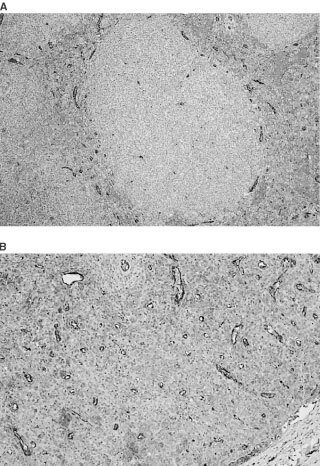
Microvessels in NHL. Representative field of follicular NHL (**A**) and diffuse NHL (**B**) stained with anti-factor-VIII-related antigen. Note the absence of microvessels in the follicular area.

Mean MVC, determined in 40 patients was 77.3±35.7 (range 20–197). Follicular lymphoma specimens demonstrated a distinct pattern of microvessel distribution, with very few microvessels in the area of the follicles but numerous ones in the areas between the follicles (Figure 1

). Since the vessels were counted in the areas of most intense vascularisation, (hot spots), in the follicular lymphomas specimens, these areas were always in the interfollicular areas. No correlation was found between mean MVC and grade, stage histology or survival of the patients.

### bFGF expression in lymphoma specimens (Figure 3A)

**Figure 3:**
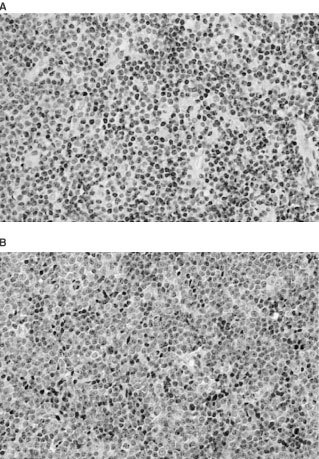
Staining of small lymphocytic lymphoma cells for bFGF (**A**), FGFR-1 (**B**).

Staining of lymphoma specimens for bFGF was performed in 39 patients. In nine patients the lymphomas expressed bFGF (23.1%). There was heterogeneity of bFGF expression with a mean staining of 51% of lymphoma cells (range 15–100%). In most specimens endothelial cells, polymorphonuclear cells, and macrophages stained positive and served as an internal positive control. Positive immunostaining for bFGF was usually present in the cytoplasm of tumour cells, but some cells showed nuclear staining. Lymphoma cell staining correlated strongly with PFS (*P*=0.003, Table 2

**Table 2:**
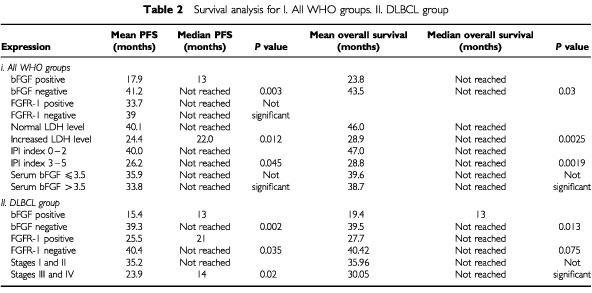
Survival analysis for I. All WHO groups. II. DLBCL group

, Figure 2A

**Figure 2:**
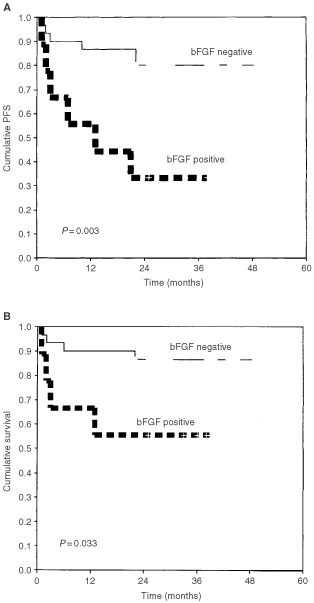
Kaplan–Meier progression-free survival (**A**) and overall survival (**B**) curves according to bFGF expression for 39 patients with NHL. bFGF expression was positive in lymphomas from nine patients (23%).

) and overall survival (*P*=0.03, Table 2, Figure 2B), with the patients showing positive staining for bFGF having worse PFS and overall survival. In the nine patients in whom the lymphoma cells stained positive, mean survival was 23.8±5.8 months (range 1–38; median survival was not reached)) and mean PFS was 17.9±5.1 months (range 1–38, median 13 months). Four of them died during the study period, and only 33% had no disease progression. By contrast, of the 30 patients whose specimens stained negative for bFGF, mean survival was 43.5±2.6 months (range 1–49, median survival was not reached) and mean PFS 41.2±2.9 months (range 1–49, median PFS was not reached). Only four patients from this group died during the study period, and 80% had no disease progression. When survival analysis were carried out in the 25 patients with diffuse large B-cell lymphoma (DLBCL), the patients in whom the lymphoma specimens stained positive for bFGF had significantly worse overall (*P*=0.013) and PFS (*P*=0.002, Table 2). There was also a correlation between bFGF staining and bulky disease. Five of the nine patients with lymphoma which was positive for bFGF staining (55.6%) had bulky disease compared to 17.9% of the patients with lymphoma with negative staining (*P*=0.04). No correlation was found between bFGF staining and serum bFGF concentration, MVC, histology, stage, grade, LDH level, IPI index, or response to treatment.

### bFGF receptor expression in lymphoma specimens (Figure 3B)

Staining of lymphoma specimens for bFGF receptor (FGFR-1) was performed in 41 patients. Results were positive in 24 (58.5%). In most of the positive specimens the staining was cytoplasmatic, but in some, additional nuclear staining or only nuclear staining was evident. Endothelial cells stained positive as did plasma cells, and they served as an internal positive control. A correlation was found between a negative expression of FGFR-1 and the achievement of complete remission (*P*=0.047). Eleven of the 17 patients with lymphomas with negative staining for FGFR-1 (64.7%) achieved complete remission compared to only eight of the 24 patients with lymphomas with positive staining (33.3%). There was also a borderline correlation between FGFR-1 expression and bulky disease (*P*=0.07), with 39% of the patients with lymphomas that expressed FGFR-1 having bulky disease compared to 12.5% of patients with lymphomas who did not. A borderline correlation was noted between the expression of bFGF and its receptor (*P*=0.067): 87.5% of the patients who expressed bFGF in their lymphomas, also expressed FGFR-1, compared to only 50% of the patients who's lymphomas did not express bFGF. No correlation was found between FGFR-1 staining and serum bFGF concentration, MVC, histology, stage, grade, LDH level, IPI index or overall survival. When survival analysis was carried out for the 24 patients with DLBCL group alone, there was a significant worse PFS for the patients with positive staining (*P*=0.035, Table 2) and borderline worse overall survival (*P*=0.075, Table 2).

### Prognostic studies

The prognosis of patients with NHL is best determined by the IPI index and this was confirmed by the present study (*P*=0.002). Multivariate analysis by Stepwise Cox regression was used to determine if bFGF expression adds to the predictive value of the IPI for survival. IPI alone still had the strongest predictive value.

## DISCUSSION

The results presented show that bFGF and its receptor FGFR-1 are expressed in NHL. Furthermore, the expression of bFGF and its receptor in lymphoma cells has a prognostic significance: bFGF expression is correlated with worse survival and PFS, and FGFR-1 expression is correlated with decreased rate of achievement of complete remission and in the subgroup with DLBCL with worse survival and PFS. Thus, bFGF and its receptor might be involved in the survival and resistance to therapy of NHL cells.

Several lines of evidence have suggested a role of fibroblast growth factors (FGFs) and their receptors (FGFRs) in human cancer (Birnbaum *et al*, 1991). Basilico and Moscatelli (1992) found that amplification or ectopic expression of FGFs induces cellular transformation. Several carcinomas have shown increased bFGF production by the cancer epithelial cell themselves observed by immunohistochemistry. These include pancreatic (Ohta *et al*, 1995; Yamazaki *et al*, 1997) breast (Yiangou *et al*, 1997; Blanckaert *et al*, 1998), non-small cell lung (Volm *et al*, 1997) and head and neck squamous carcinomas (Dellacono *et al*, 1997). FGFR-1 and FGFR-2 are overexpressed in several human cancers (Birnbaum *et al*, 1991; Ohta *et al*, 1995; Yamazaki *et al*, 1997; Blanckaert *et al*, 1998), and some investigators noted a prognostic significance of bFGF expression or bFGF receptors only. In pancreatic carcinomas, expression of bFGF was strongly associated with the tumour cell proliferation (Yamazaki *et al*, 1997) and with poor prognosis. Ohta *et al* (1995) reported that increased FGFR-1 expression in pancreatic carcinomas was correlated with decreased survival. In non-small cell lung carcinomas, all tumour specimens expressed some level of bFGF and FGFR-1 (Volm *et al*, 1997). Patients with high FGFR-1 expression had significantly shorter survival than patients with weak or moderate expression, but no correlation was found between bFGF expression and patient survival. In patients with breast carcinomas, higher levels of bFGF levels were associated with improved overall and disease-free survival (Yiangou *et al*, 1997); However, bFGF was expressed less in the malignant tissue than the non-malignant breast tissue. In the study of Blanckaert *et al* (1998) almost all breast tumours contained high-affinity bFGFR, and the patients expressing high levels of bFGFR had a more favourable prognosis. In prostate cancer, Giri *et al* (1999) found that bFGF is significantly increased relative to the normal prostate tissue and that it is localised in stromal fibroblasts and endothelial cells but not malignant cells. In a subset of prostate cancers, however, these authors observed overexpression of both FGFR-1 and FGFR-2 in the epithelial cells, which correlated with poor differentiation.

Recently, intracellular bFGF has been detected in several lymphoproliferative diseases and was associated with more advanced or refractory disease. In B cells derived from chronic lymphocytic leukaemia, elevated levels of intracellular levels were correlated with disease stage and were associated with resistance to fludarabine (Menzel *et al*, 1996). Gruber *et al* (1999) showed that in hairy cell leukaemia, another type of chronic B-cell leukaemia, the leukemic cells express bFGF, which in turn, may mediate the resistance to chemotherapy and survival of the malignant cells. Vacca *et al* (1999) were the first to demonstrate a significant increase in bone marrow angiogenesis (evaluated as microvessel area) in patients with active multiple myeloma (MM) compared with patients with nonactive MM and monoclonal gammopathy of undetermined significance (MGUS). Evaluation of bFGF in plasma cell lysates by immunoassay showed significantly higher levels in cells of the patients with active MM compared with nonactive MM and MGUS patients. However, when all the patients were considered, there was no significant correlation between individual plasma cell bFGF levels and bone marrow neovascularisation. It has recently been reported that patients with MM who respond to chemotherapy show a significant decrease in serum bFGF levels, whereas nonresponders do not (Sezer *et al*, 2001).

In the present study, we did not detect a significant change in serum bFGF levels after 2–3 cycles of chemotherapy, nor did we find a correlation between MVC and NHL histology or grade or between MVC and prognosis. One explanation might be the microvessel counting method used. We did observe a lack of microvessels in the follicular areas of follicular lymphoma (Figure 1) but there were many microvessels in the interfollicular areas, and those areas with the greatest number of microvessels (‘hot spots’) were counted.

Unlike Salven *et al* (1999), we did not find that pretreatment serum bFGF level is associated with poor overall survival. This difference might be attributable to the relatively small number of patients in our study. Nevertheless, the sample was large enough to yield a highly significant correlation between bFGF expression and poor PFS and overall survival. We also found that the MVC did not correlate with the expression of bFGF, the expression of its receptor, or patient survival. This might suggest that the autocrine or paracrine loop involving the lymphoma cells is more important in NHL than the paracrine loop involving the endothelial cells.

Beside autocrine loop activation, lymphoma cells expressing bFGFRs may respond to bFGFs produced by other cell types or released from the extracellular matrix in a paracrine fashion. We speculate that as the tumour becomes more aggressive, it also becomes independent of stromal paracrine factors by the establishment of an autocrine FGF stimulation that can increase its tumorigenicity.

Our results suggest that bFGF within the lymphoma cells plays an important role in the pathogenesis of NHL and can identify patients with poor outcome. The present study is small and heterogeneous. Further larger studies are required examining specific types of lymphoma to determine in what subtypes the effect is most significant. If true, agents that can suppress bFGF synthesis might have a role in the treatment of resistant NHL. Strategies aimed at decreasing the expression of bFGF and its receptor may be of therapeutic benefit in poor-prognosis NHL.
